# Precise sound characteristics drive plasticity in the primary auditory cortex with VNS-sound pairing

**DOI:** 10.3389/fnins.2023.1248936

**Published:** 2023-09-05

**Authors:** Michael S. Borland, Elizabeth P. Buell, Jonathan R. Riley, Alan M. Carroll, Nicole A. Moreno, Pryanka Sharma, Katelyn M. Grasse, John M. Buell, Michael P. Kilgard, Crystal T. Engineer

**Affiliations:** ^1^Department of Neuroscience, School of Behavioral and Brain Sciences, The University of Texas at Dallas, Richardson, TX, United States; ^2^Texas Biomedical Device Center, The University of Texas at Dallas, Richardson, TX, United States; ^3^Erik Jonsson School of Engineering and Computer Science, The University of Texas at Dallas, Richardson, TX, United States

**Keywords:** neuromodulation, vagus nerve stimulation, plasticity, auditory cortex, speech

## Abstract

**Introduction:**

Repeatedly pairing a tone with vagus nerve stimulation (VNS) alters frequency tuning across the auditory pathway. Pairing VNS with speech sounds selectively enhances the primary auditory cortex response to the paired sounds. It is not yet known how altering the speech sounds paired with VNS alters responses. In this study, we test the hypothesis that the sounds that are presented and paired with VNS will influence the neural plasticity observed following VNS-sound pairing.

**Methods:**

To explore the relationship between acoustic experience and neural plasticity, responses were recorded from primary auditory cortex (A1) after VNS was repeatedly paired with the speech sounds ‘rad’ and ‘lad’ or paired with only the speech sound ‘rad’ while ‘lad’ was an unpaired background sound.

**Results:**

Pairing both sounds with VNS increased the response strength and neural discriminability of the paired sounds in the primary auditory cortex. Surprisingly, pairing only ‘rad’ with VNS did not alter A1 responses.

**Discussion:**

These results suggest that the specific acoustic contrasts associated with VNS can powerfully shape neural activity in the auditory pathway. Methods to promote plasticity in the central auditory system represent a new therapeutic avenue to treat auditory processing disorders. Understanding how different sound contrasts and neural activity patterns shape plasticity could have important clinical implications.

## Introduction

Some sounds are acoustically very similar and are hard to tell apart perceptually, while other sounds are acoustically very distinct and are easy to behaviorally discriminate. For example, the English consonants ‘D’ and ‘B’ have distinct acoustics and evoke distinct neural activity patterns, while the consonants ‘R’ and ‘L’ have similar acoustics and evoke very similar neural activity patterns in both human and rodent brains (Engineer et al., 2008). Speech sounds with similar acoustics, like ‘R’ and ‘L’, are often difficult contrasts for foreign language learners to master (Logan et al., 1991; Callan et al., 2003; Kuhl, 2004). Interestingly, non-native English speakers have more similar neural activity patterns evoked by the sounds ‘R’ and ‘L’ compared to native English speakers, who have more distinct neural activity patterns evoked by the sounds ‘R’ and ‘L’ (Raizada et al., 2010). Rats, like foreign language learners, have difficulty learning to discriminate between the ‘R’ and ‘L’ sounds, and weeks of extensive training fails to improve both their behavioral and neural accuracy on this task (Engineer et al., 2015b). Similarly, in humans, extensive discrimination training does not result in neural activity patterns that are more distinct (Callan et al., 2003). Developing a technique that can result in more distinct neural activity patterns and more easily discriminable sounds would be useful to multiple clinical populations with receptive language deficits, including individuals with autism spectrum disorder, or stroke survivors (Raizada et al., 2010; Engineer et al., 2017a).

Pairing sounds with neuromodulator release can enhance cortical responses to the paired sounds (Kilgard and Merzenich, 1998; Ma and Suga, 2003; Froemke et al., 2007; Edeline et al., 2011; Engineer et al., 2011; Martins and Froemke, 2015). For example, pairing a 9 kHz sound with neuromodulator release significantly increases the percentage of auditory cortex that can respond to the paired sound frequency (Engineer et al., 2011; Borland et al., 2015, 2019). Similarly, pairing the sounds ‘rad’ and ‘lad’ with neuromodulator release, through vagus nerve stimulation (VNS) 300 times per day for 20 days, strengthens the primary auditory cortex (A1) response strength to both ‘rad’ and ‘lad’ in typically hearing rats (Engineer et al., 2015a). Although the neural response to the paired sounds is stronger, a stronger response is not necessarily more discriminable, and it is not yet known whether VNS-sound pairing results in neural responses that are more discriminable (Raizada et al., 2010). In this set of experiments, we test the hypothesis that pairing VNS with sounds will make the primary auditory cortex responses to the paired sounds more distinct.

## Materials and methods

Twenty four adult female Sprague–Dawley rats were used for these experiments. Six rats served as experimentally naïve control rats, and 18 rats experienced VNS-sound pairing. All experimental protocols were approved by The University of Texas at Dallas Institutional Animal Care and Use Committee (IACUC).

### VNS surgery

Rats were implanted with a cuff electrode around the vagus nerve as well as a headcap used to connect the electrode leads to the stimulator. The bipolar cuff electrode was constructed using the methods and materials described in our detailed published protocol (Rios et al., 2019). The surgical procedures to implant the cuff electrode around the left vagus nerve were identical to previous studies (Engineer et al., 2011, 2015a; Borland et al., 2015, 2018, 2019). First, rats were anesthetized with ketamine hydrochloride (80 mg/kg) and xylazine (10 mg/kg). Lidocaine was injected before all incisions. An incision was made in the neck to expose the left vagus nerve, and a second incision was made at the top of the head to expose the skull. The cuff electrode was wrapped around the nerve, and leads from the electrode were subcutaneously tunneled between the left eye and left ear to the top of the skull. A custom-made Omnetics headcap was attached to the skull with bone screws and acrylic, and leads from the electrode were attached to the headcap. All incisions were sutured and covered with antibiotic ointment. All rats had 1 week of recovery following surgery.

### Acoustic properties of the speech sounds

**Figure 1:**
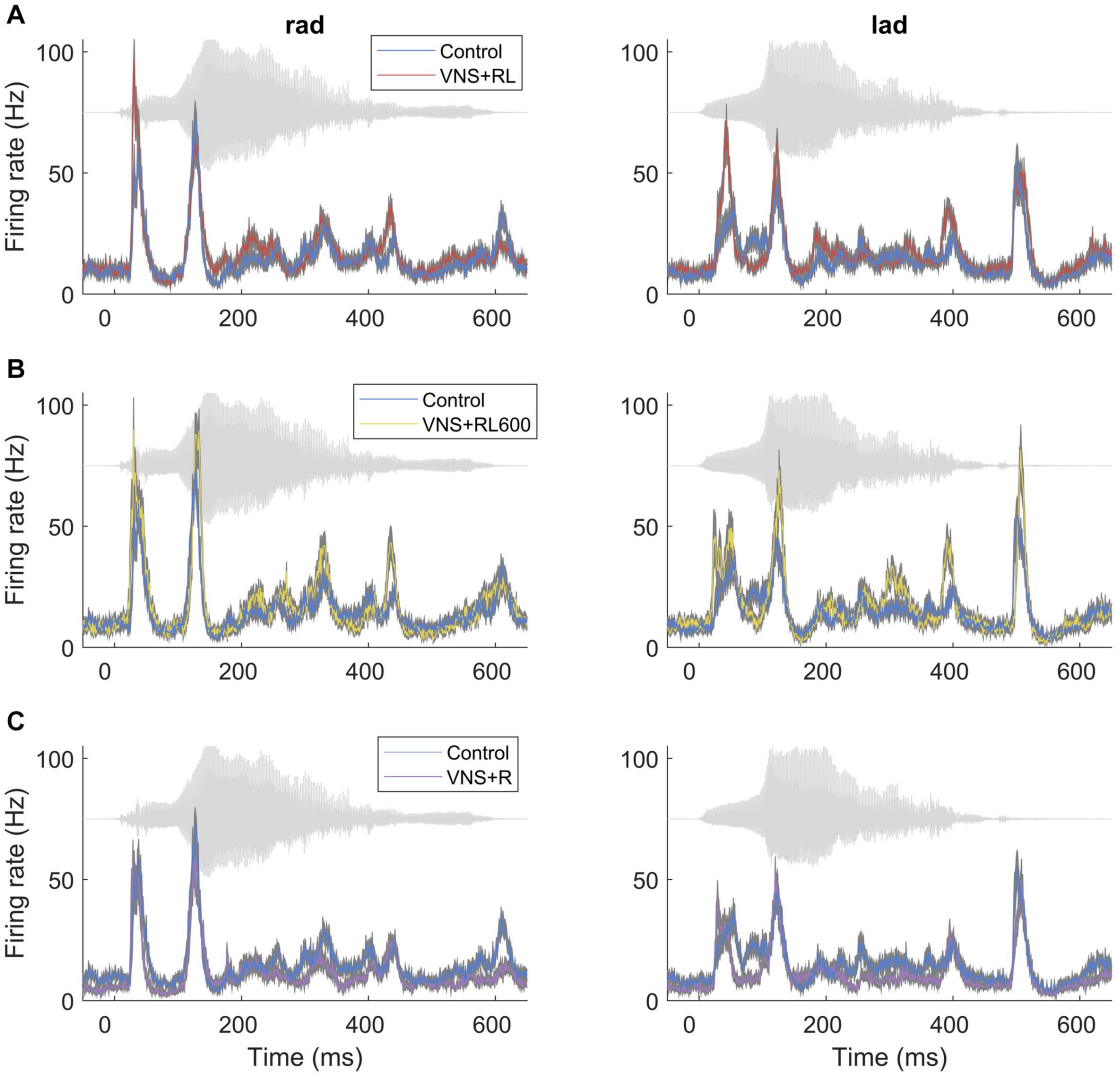
VNS paired with the sounds ‘rad’ and ‘lad’ increases the primary auditory cortex response strength to the paired sounds. PSTH primary auditory cortex responses to the paired sounds ‘rad’ and ‘lad’ in experimentally naïve rats and rats that experienced **(A)** VNS + RL pairing, **(B)** VNS + RL600 pairing, and **(C)** VNS + R pairing. The acoustic waveform in each sound is presented behind each PSTH in gray.

The sounds ‘rad’ and ‘lad’ were spoken by a female native English speaker and were approximately 600 ms in duration. The waveforms of each sound are presented in gray in Figure 1. These sounds are identical to our previous studies, and the spectrograms, amplitude envelopes, and power spectrums for these sounds are provided in these studies (Engineer et al., 2008, 2015a). The sounds ‘rad’ and ‘lad’ were presented at an intensity of 60 dB, using the loudest 100 ms of the vowel portion of the sound. As a result, the consonant onset portion of each sound was approximately 40 dB. Both sounds were spectrally shifted up by one octave into the rat hearing range using the STRAIGHT vocoder. The sounds /r/ and /l/ differ in their third formant values, where /r/ has lower F3 values, and /l/ has higher F3 values (Raizada et al., 2010).

### VNS-speech pairing

Following recovery, rats underwent VNS-sound pairing for 20 days (Table 1). Six rats experienced VNS presented with the sound ‘rad’ while the background sound ‘lad’ was not paired with VNS for 300 VNS-sound pairings per day (VNS + R group). Eight rats experienced VNS presented with the sounds ‘rad’ and ‘lad’ for 300 VNS-sound pairings per day (VNS + RL group). Four rats experienced VNS presented with the sounds ‘rad’ and ‘lad’ for 600 VNS-sound pairings per day (VNS + RL600 group). Six rats were experimentally naïve control rats that did not experience VNS-sound pairing (Control group). No rats were exposed to the sounds alone, without VNS pairing. All rats were awake and able to freely move around the booth during the entirety of the VNS-sound pairing sessions. Other than the sounds presented, all VNS-sound pairing methods were identical to previous studies (Engineer et al., 2011, 2015a; Borland et al., 2019). Briefly, the 500 ms duration pulse train of VNS onset occurred 50 ms before the sound onset. The VNS was presented at an intensity of 0.8 mA, a frequency of 30 Hz, and a biphasic pulse width of 100 μs. The timing of each VNS-speech pairing trial was randomized so the rats could not predict when pairing would occur, with an average of 30 s between pairing trials.

**Table 1:** The number of VNS-sound pairings experienced per day for each sound for each experimental group.

Experimental group	Rad	Lad
Naïve control	–	–
VNS + R	300 VNS-paired	300 not paired
VNS + RL	150 VNS-paired	150 VNS-paired
VNS + RL600	300 VNS-paired	300 VNS-paired

### Neural recordings

Twenty four hours after the last VNS-sound pairing session, neurophysiology recordings were collected. Responses were recorded from the primary auditory cortex (A1) from 204 recording sites in 6 experimentally naïve control rats, 207 recording sites from 6 VNS + R paired rats, 264 recording sites from 8 VNS + RL paired rats, and 156 recording sites from 4 VNS + RL600 paired rats. Rats were anesthetized with sodium pentobarbital (50 mg/kg), and they received supplemental doses regularly as needed. Body temperature, heart rate, and blood oxygenation were monitored throughout the experiment. All rats regularly received a 1:1 mix of Ringers lactate and dextrose. Rats received a tracheostomy to facilitate breathing and a cisternal drain to prevent brain swelling. For primary auditory cortex recordings, a craniotomy was performed over the right auditory cortex. The dura was resected and pairs of Parylene-coated tungsten microelectrodes (1–2 MΩ, FHC) were lowered into the brain. At each recording site, tones and speech sounds were presented. Tones were presented at frequencies spanning 1–48 kHz in 0.0625 octave steps at intensities spanning 0–75 dB in 5 dB steps. The speech sounds ‘bad’, ‘dad’, ‘deed’, ‘dood’, ‘gad’, ‘lad’, ‘mad’, ‘pad’, ‘rad’, ‘sad’, and ‘tad’ were presented. All speech sounds were presented so that the loudest 100 ms of the vowel portion of the sound was 60 dB, and shifted up in frequency by 1 octave using the STRAIGHT vocoder to better match the rat hearing range (Engineer et al., 2008). Speech sounds were each presented 20 times and were randomly interleaved at each recording site. Tucker Davis Technologies (TDT) hardware and software were used to present sounds and record neural responses. All neural recording procedures were identical to the procedures used in previous studies (Borland et al., 2019).

### Data analysis

Data analysis was performed in MATLAB and SPSS version 28. The driven firing rate response to speech sounds was quantified as the sum of the number of spikes evoked in the first 40 ms of the onset response to the sound minus the spontaneous firing rate recorded before sound onset. Neural detection and discrimination accuracy was quantified using a nearest-neighbor classifier (Engineer et al., 2008, 2015a). Neural discrimination accuracy using this classifier is highly correlated with rat behavioral sound discrimination accuracy (Engineer et al., 2008; Perez et al., 2013). For example, sounds that evoke very similar neural activity patterns, like ‘rad’ and ‘lad’, have low neural and behavioral discrimination accuracy, while sounds that evoke very distinct neural activity patterns, like ‘dad’ and ‘sad’, have high neural and behavioral discrimination accuracy. For each recording site, an average sound template was constructed for the sound ‘rad’ and the sound ‘lad’ using 19 of the 20 recorded repeats of the response to the 40 ms onset portion of the sound, with 1 ms precision. The remaining single trial repeat was compared to the average sound templates and was assigned to the sound template that it most closely resembled, using Euclidean distance. The classifier evaluated neural activity pattern evoked by the sound ‘rad’ vs. the neural activity pattern evoked by the sound ‘lad’. The classifier used a leave-one-out design, where for each recording site, the classifier was run 40 times using a different training and testing set each time (the 20 repeats of the response to ‘rad’ and the 20 repeats of the response to ‘lad’ were each individually used as a test trial, with the remaining repeats used as training templates). Classifier accuracy was quantified as the percentage of trials that were correctly assigned, where chance performance for neural detection and discrimination accuracy is 50%. All speech response strength and neural classifier statistical comparisons used Kruskal-Wallis non-parametric tests with post-hoc pairwise comparisons with Bonferroni correction for multiple comparisons. Figures plot median values along with 95% bootstrap confidence intervals using 50,000 bootstrap samples.

The response strength to tones was quantified in one-octave bins, from 1 to 2, 2 to 4, 4 to 8, 8 to 16, and 16 to 32 kHz. A generalized linear mixed-effects model was used to account for the different number of A1 recording sites obtained in each rat, using a linear model with an identity link. The experimental group and tone frequency were evaluated as fixed factors, and the rat was evaluated as a random factor. Robust estimation was used to account for any potential variations of model assumptions. *Post hoc* comparisons used simple contrasts to compare each VNS-sound paired group to the experimentally naïve control group.

For each rat, maps of primary auditory cortex were constructed using the Voronoi tessellation procedure, as in previous studies (Recanzone et al., 1993; Kilgard and Merzenich, 1998; Engineer et al., 2004; Yokota et al., 2015). Using the tessellation procedure, polygons are generated from recording site locations that are not uniformly spaced, so that each point in a given polygon is closer to the recording site location at the polygon center than any other recording site location. The primary auditory cortex area was quantified by taking the sum of the area of all of the individual polygons representing A1 recording sites. The A1 area between groups was compared using a one-way ANOVA.

Receptive field properties were also quantified based on the evoked response to the same 25 ms tones used to quantify the response strength to tones. These receptive field properties include response threshold, bandwidths 10–40 dB above the response threshold, onset latency, peak latency, end of peak latency, and spontaneous firing rate. The response threshold was defined as the minimum sound intensity that evoked a driven response at a recording site’s characteristic frequency. Bandwidths were defined as the range of driven activity evoked 10–40 dB above the response threshold. The onset latency was defined as the time point when the firing rate was more than 2 standard deviations above the spontaneous firing rate. The end of peak latency was defined as the time point when the firing rate went below 2 standard deviations above the spontaneous firing rate. The peak latency was defined as the time point of maximal firing between the onset and end of peak latencies. All receptive field statistical comparisons used Kruskal-Wallis non-parametric tests with *post-hoc* pairwise comparisons with Bonferroni correction for multiple comparisons.

## Results

### VNS + RL increases the primary auditory cortex response strength to the paired sounds

**Figure 2:**
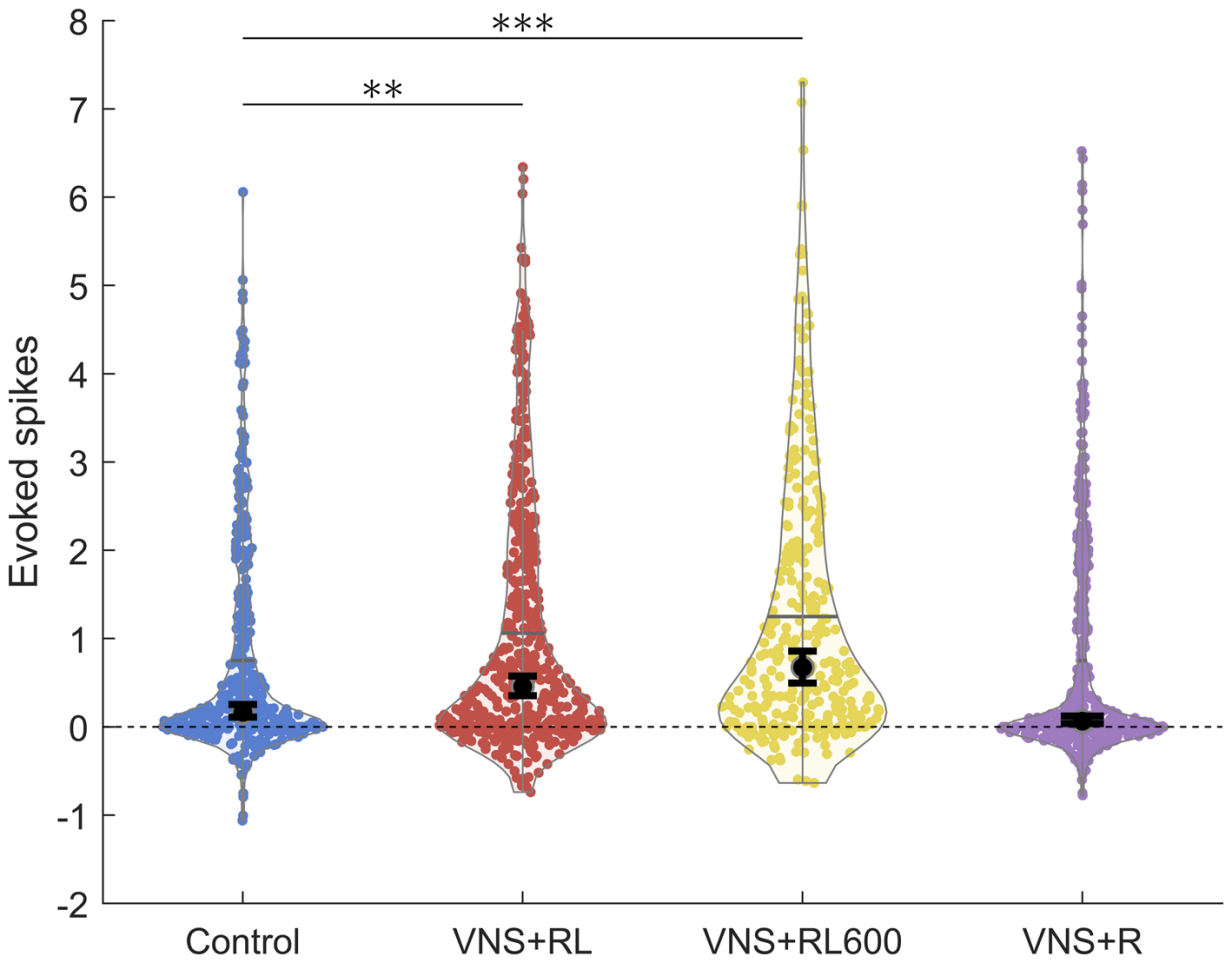
VNS + RL pairing significantly alters primary auditory cortex responses. A1 responses to the paired sounds are stronger following VNS + RL and VNS + RL600 pairing. Black dots indicate median responses for each group, and gray lines indicate mean responses for each group. Error bars indicate 95% bootstrap confidence intervals of the median. Asterisks indicate experimental groups that are statistically significant from the control group. *p < 0.05; **p < 0.01; ***p < 0.001.

We have previously documented that VNS paired with the speech sounds ‘rad’ and ‘lad’ selectively increases the primary auditory cortex response to the paired sounds (Engineer et al., 2015a). In this study, we observed a significant effect of experimental group on the primary auditory cortex response strength to the paired sounds (χ^2^(3) = 56.1, *p* < 0.001). We first replicated our previous findings using the same VNS-sound pairing parameters and paired ‘rad’ and ‘lad’ with VNS 300 times per day for 20 days. VNS-speech pairing significantly increased the A1 response strength to the paired speech sounds ‘rad’ and ‘lad’ compared to the experimentally naïve control group (*p* = 0.009, Figures 1A, 2). Compared to experimentally naïve rats, the paired speech sounds evoked more spikes in rats who experienced 300 VNS-speech pairings per day.

Next, we investigated whether providing twice as many VNS-sound pairing stimulations per day would impact primary auditory cortex responses to the paired sounds. In this experiment, we paired ‘rad’ and ‘lad’ with VNS 600 times per day for 20 days. Compared to experimentally naïve rats, the paired speech sounds evoked significantly more spikes in rats who experienced 600 VNS + RL pairings per day (*p* < 0.001, Figures 1B, 2). There was no difference in evoked spikes between rats that experienced 300 vs. 600 VNS-speech pairings per day (*p* = 0.09, Figure 2).

In the next experiment, we investigated whether pairing VNS with one sound but not the other sound would impact primary auditory cortex responses to the paired sounds, with the goal of making the sounds more discriminable. In this experiment, we paired ‘rad’ with VNS 300 times per day, while 300 presentations of ‘lad’ were interleaved but not paired with VNS. Compared to experimentally naïve control rats, the response strength to the paired speech sounds was unaltered in rats who experienced VNS paired with ‘rad’ but not ‘lad’ (*p* = 0.46, Figures 1C, 2). The response strength to the paired sounds was significantly weaker in rats who experienced VNS paired with ‘rad’ but not ‘lad’ compared to rats who received both ‘rad’ and ‘lad’ with VNS 300 times per day (*p* = 0.009) and 600 times per day (*p* < 0.001).

### VNS + RL increases neural detection ability

**Figure 3:**
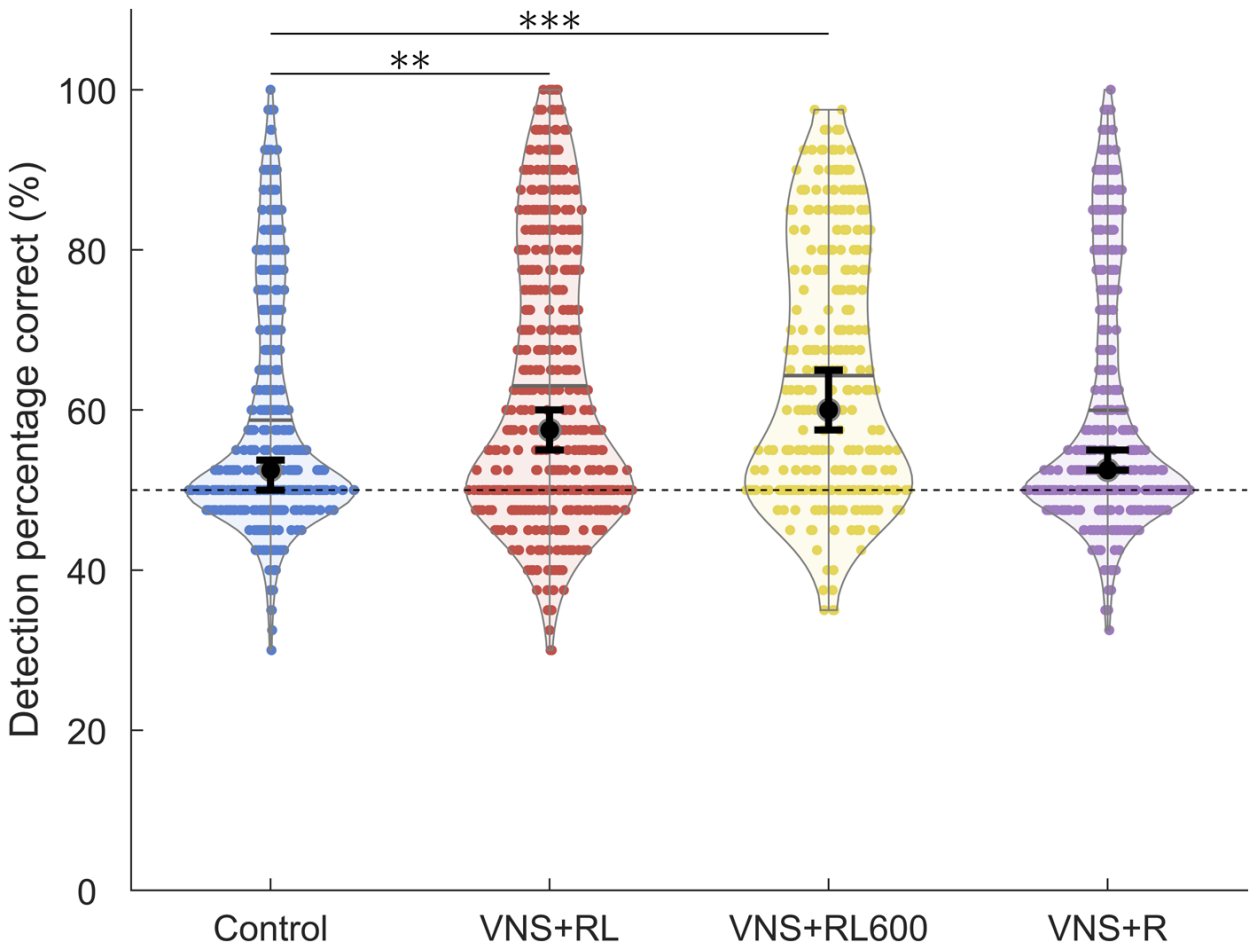
VNS + RL pairing significantly alters neural detection accuracy. A1 responses to the paired sounds are more detectable following both 300 or 600 VNS + RL pairings per day. Black dots indicate median responses for each group, gray lines indicate mean responses for each group, and dashed lines indicate neural classifier chance performance (50% correct). Error bars indicate 95% bootstrap confidence intervals of the median. Asterisks indicate experimental groups that are statistically significant from the control group, *p < 0.05; **p < 0.01; ***p < 0.001.

We next examined the ability of a nearest-neighbor classifier to detect the primary auditory cortex activity patterns evoked by the paired sounds ‘rad’ and ‘lad’. We observed a significant effect of experimental group on the primary auditory cortex neural detection accuracy of the paired sounds [*χ*^2^(3) = 32.1, *p* < 0.001]. The nearest-neighbor neural classifier was better able to detect the paired sounds ‘rad’ and ‘lad’ in the VNS + RL paired rats compared to experimentally naïve control rats (*p* = 0.001, Figure 3). In experimentally naïve control rats, the classifier was able to detect A1 neural activity patterns evoked by the paired sounds with a median of 52.5% accuracy. Classifier accuracy was significantly enhanced in the rats who experienced 300 VNS-speech pairings per day to a median of 57.5% accuracy.

Classifier detection accuracy was also significantly enhanced in rats who experienced 600 VNS-speech pairings per day compared to experimentally naïve control rats (*p* < 0.001, median of 60% accuracy, Figure 3). However, there was no significant difference in classifier detection accuracy between rats that experienced 300 vs. 600 VNS-RL pairings per day (*p* = 0.55, Figure 3).

Similar to the response strength, classifier detection accuracy was unaltered in rats who experienced VNS paired with ‘rad’ but not ‘lad’ compared to experimentally naïve control rats (*p* = 1, median of 52.5% accuracy, Figure 3). Classifier detection accuracy of the paired sounds was significantly less accurate in rats who experienced VNS paired with ‘rad’ but not ‘lad’ compared to rats who received both ‘rad’ and ‘lad’ with VNS 300 times per day (*p* = 0.04) and 600 times per day (*p* < 0.001).

### VNS + RL increases neural discriminability

**Figure 4:**
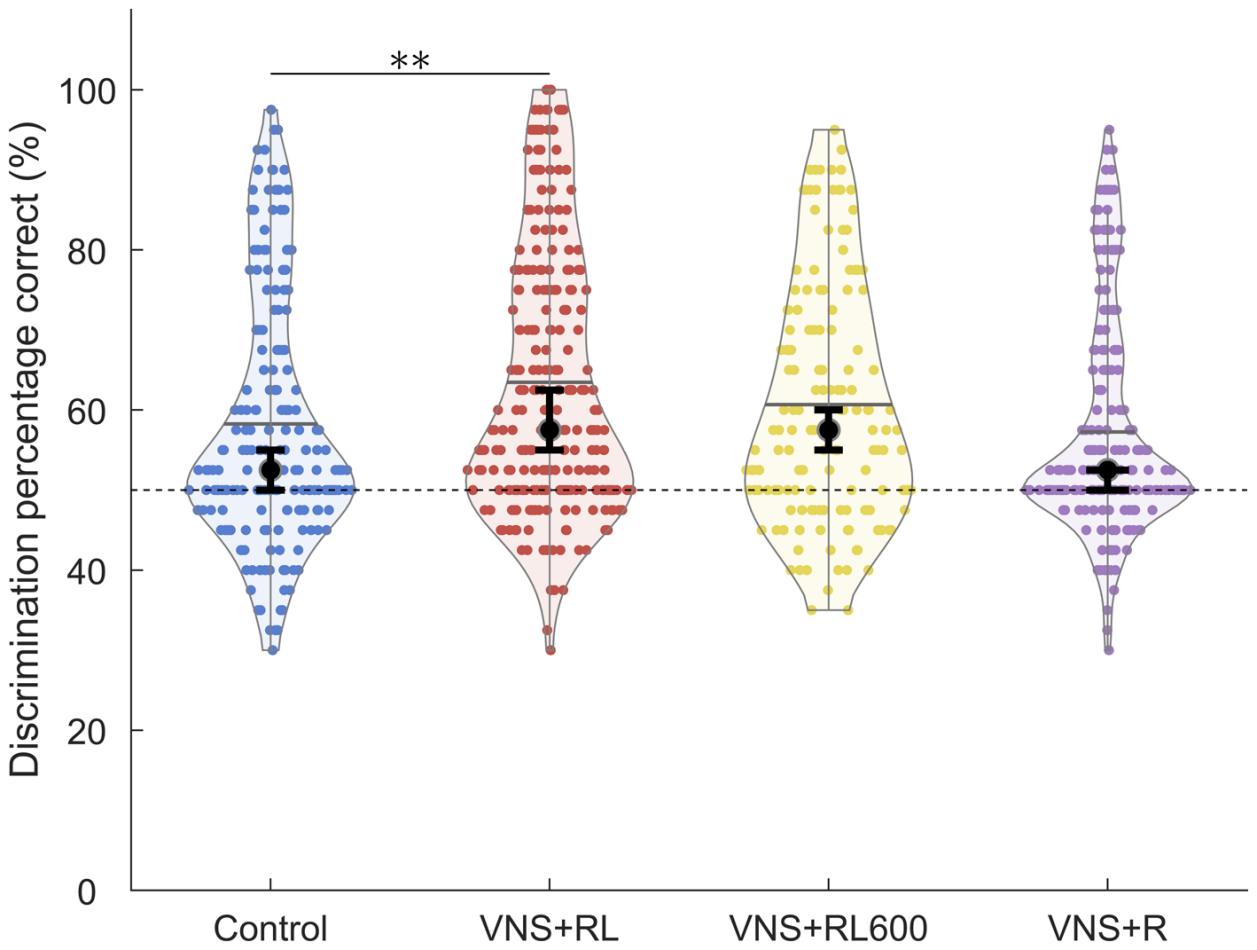
VNS + RL pairing significantly alters neural discrimination accuracy. A1 responses to the paired sounds are more discriminable following 300 VNS + RL pairings per day, but not 600 VNS + RL pairings per day or following VNS + R pairing. Black dots indicate median responses for each group, gray lines indicate mean responses for each group, and dashed lines indicate neural classifier chance performance (50% correct). Error bars indicate 95% bootstrap confidence intervals of the median. Asterisks indicate experimental groups that are statistically significant from the control group, *p < 0.05; **p < 0.01; ***p < 0.001.

**Figure 5:**
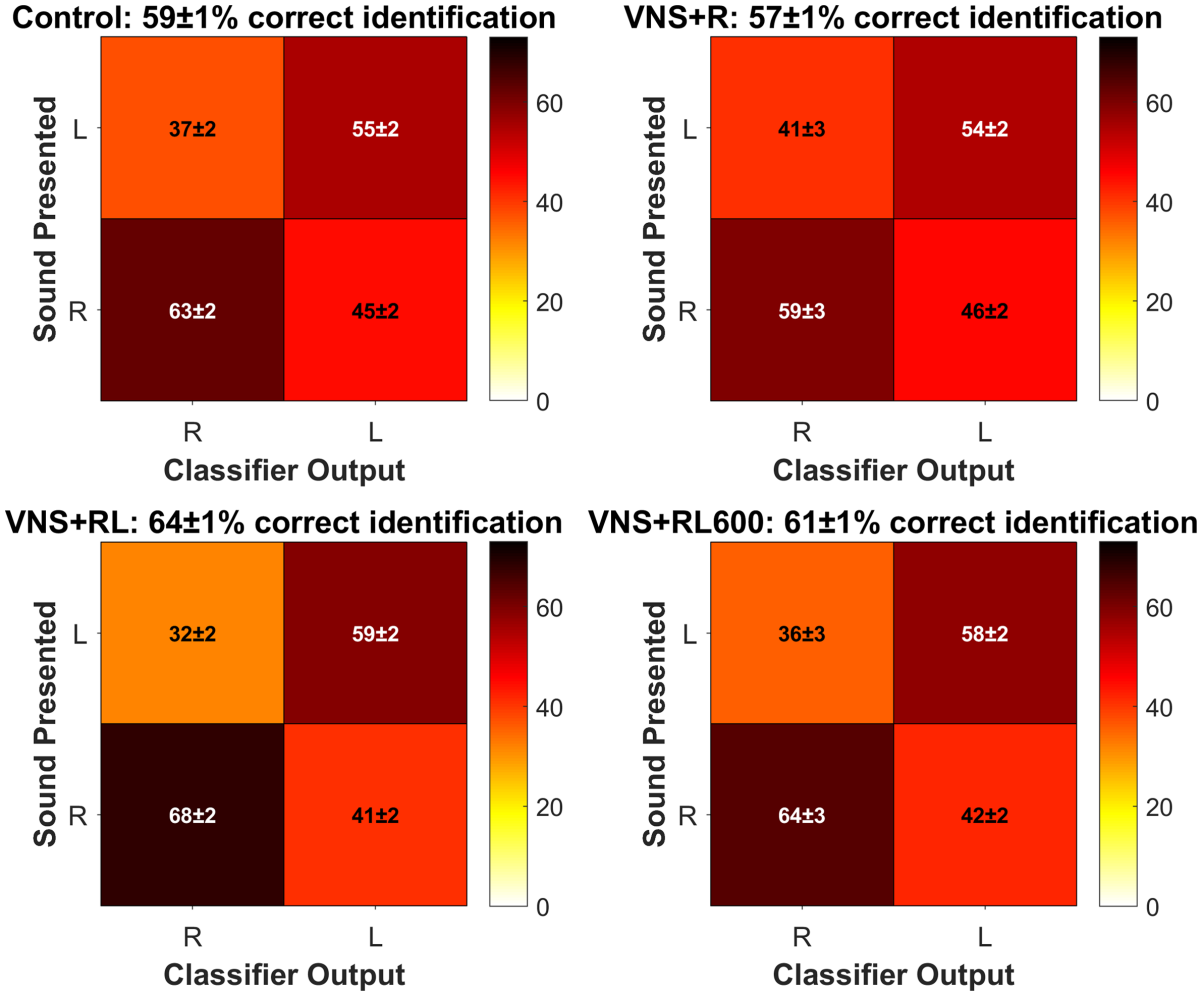
Confusion matrices plotting classifier R vs. L accuracy for each group. The sound presented is plotted on the y axis, and the classifier guess is plotted on the x axis. The color bar indicates the percentage of trials where the classifier assigned the trial to the R or L category. White font indicates the mean ± SEM percentage of trials that the classifier correctly assigned to the correct category, while black font indicates the mean ± SEM percentage of trials that the classifier incorrectly assigned to the wrong category. For each group, the mean ± SEM classifier accuracy is given in the subplot title. There were no significant differences between experimental groups for the percentage of trials that the classifier correctly assigned R when the sound presented was R [χ^2^(3) = 6.804, *p* = 0.078, Kruskal-Wallis non-parametric test] or that the classifier correctly assigned L when the sound presented was L [χ^2^(3) = 3.722, *p* = 0.293].

We next examined the ability of a nearest-neighbor classifier to discriminate between the primary auditory cortex activity patterns evoked by the paired sounds ‘rad’ versus ‘lad’. We observed a significant effect of experimental group on the primary auditory cortex neural discrimination accuracy of the paired sounds [*χ*^2^(3) = 20.5, *p* < 0.001]. The nearest-neighbor neural classifier was better able to discriminate between A1 activity patterns evoked by ‘rad’ compared to patterns evoked by ‘lad’ in the VNS + RL paired rats compared to experimentally naïve control rats (*p* = 0.003, Figures 4, 5). Classifier discrimination accuracy was significantly increased in rats who experienced 300 VNS-speech pairings per day (median of 57.5% [95% confidence interval of 55–62.5%] vs. 52.5% [95% confidence interval of 50–55%]). These findings successfully replicate and expand upon our previous findings (Engineer et al., 2015a) and document that VNS paired with speech sounds enhances the A1 response strength and discriminability of the paired sounds.

Interestingly, classifier discrimination accuracy was not significantly different between rats who experienced 600 VNS-speech pairings per day (median of 57.5% [95% confidence interval of 55–60%]) compared to experimentally naïve control rats (*p* = 0.51, Figures 4, 5) or between rats that experienced 300 vs. 600 VNS-speech pairings per day (*p* = 0.96, Figure 4). While rats who experienced 600 VNS-speech pairings per day had stronger responses to the paired sounds, the increased response strength did not make the paired sounds more discriminable from each other.

Finally, classifier discrimination accuracy was unaltered in rats who experienced VNS paired with ‘rad’ but not ‘lad’ (median of 52.5% [95% confidence interval of 50–52.5%]) compared to experimentally naïve control rats (*p* = 1, Figures 4, 5). This surprising finding indicates that both sounds need to be paired with VNS 300 times per day in order to enhance A1 response strength and discriminability of the paired sounds.

### The primary auditory cortex response strength to tones

**Figure 6:**
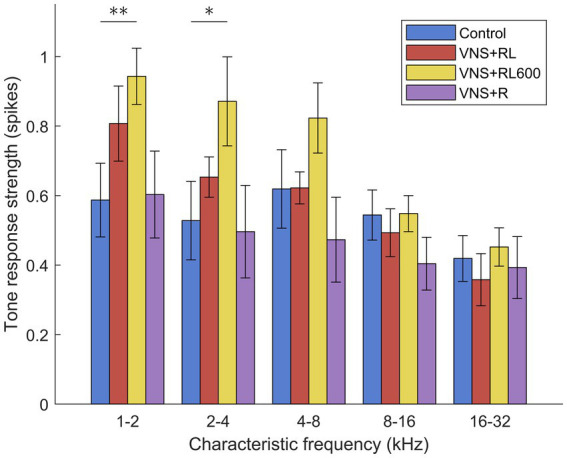
VNS + RL600 pairing significantly strengthens A1 activity evoked by low frequency tones. VNS + RL pairing and VNS + R pairing do not alter A1 activity evoked by tones. Values plotted are estimated marginal means and standard error. Asterisks indicate experimental groups that are statistically significant from the control group, *p < 0.05; **p < 0.01; ***p < 0.001.

In addition to the speech sounds paired with VNS, we also recorded A1 responses to tones to determine whether the A1 response strength changes were specific to the paired sounds. There was not a significant effect of experimental group on the primary auditory cortex response strength to tones [*F*(3, 4,135) = 2.29, *p* = 0.08]. However, there was a significant interaction between experimental group and tone frequency [*F*(12, 4,135) = 13.3, *p* < 0.001, Figure 6]. Compared to experimentally naïve control rats, the low frequency tones evoked significantly more spikes in rats who experienced 600 VNS + RL pairings per day, for tones between 1 and 2 kHz (*p* = 0.008) and for tones between 2 and 4 kHz (*p* = 0.045, Figure 6). Rats who experienced 300 VNS + RL pairings per day and rats who experienced VNS + R pairing did not exhibit any response strength changes to tones compared to experimentally naïve control rats.

### Primary auditory cortex receptive fields

Previous studies pairing VNS with a specific tone frequency have documented an increase in the percentage of A1 sites tuned to the paired tone frequency (Engineer et al., 2011; Borland et al., 2019). In this study, there was no difference between experimental groups in the percentage of primary auditory cortex sites tuned to one-octave tone frequency bins [*F*(3, 20) = 2.03, *p* = 0.14, two-way repeated measures ANOVA]. This indicates that unlike VNS-tone pairing, pairing VNS with speech sounds does not alter the characteristic frequency tuning of individual recording sites.

Similarly, previous studies have documented alterations in the total area of primary auditory cortex following training or sound pairing with neuromodulator release (Kilgard et al., 2001; Takahashi et al., 2011). In this study, there was no difference between experimental groups in the total primary auditory cortex area [*F*(3, 20) = 1.12, *p* = 0.37, one-way ANOVA]. Pairing VNS with speech sounds does not alter the size of A1.

Previous studies have also documented alterations in receptive field properties following VNS-sound pairing. In this study, we found no difference between experimental groups in the response threshold [*χ*^2^(3) = 1.8, *p* = 0.62, Table 2], onset response latency [*χ*^2^(3) = 6.6, *p* = 0.09], or peak response latency [*χ*^2^(3) = 5.7, *p* = 0.13]. The end of peak latency was significantly different between experimental groups [*χ*^2^(3) = 33.6, *p* < 0.001, Table 2]. The end of peak latency was significantly longer in VNS + RL (*p* = 0.0003), VNS + RL600 (*p* < 0.0001), and VNS + R (*p* = 0.004) rats compared to experimentally naïve control rats. Interestingly, the response bandwidth 20 and 30 dB above the response threshold was also significantly different between experimental groups [BW20: *χ*^2^(3) = 23, *p* < 0.001; BW30: *χ*^2^(3) = 25.8, *p* < 0.001, Table 2]. Response bandwidths were significantly narrower in VNS + R rats compared to experimentally naïve control rats for bandwidths 20 (*p* = 0.04) and 30 dB (*p* = 0.01) above response threshold. Finally, the spontaneous firing rate was significantly different between experimental groups [*χ*^2^(3) = 32.7, *p* < 0.001, Table 2]. The spontaneous firing rate was significantly lower in VNS + R rats compared to experimentally naïve control rats (*p* = 0.0003).

**Table 2:** Receptive field properties were altered following VNS paired with speech sounds.

	Control		VNS + RL		VNS + RL600		VNS + R	
	Mean ± SE	Median	Mean ± SE	Median	Mean ± SE	Median	Mean ± SE	Median
Threshold (dB)	18.2 ± 1.1	15	18.5 ± 0.8	20	18.6 ± 1.0	17.5	19.3 ± 0.9	20
BW10 (octaves)	1.02 ± 0.04	0.94	0.98 ± 0.04	0.88	1.17 ± 0.06	1.0	0.91 ± 0.04	0.81
BW20 (octaves)	1.46 ± 0.06	1.38	1.41 ± 0.05	1.31	1.65 ± 0.07	1.53	1.26 ± 0.05	1.06*
BW30 (octaves)	1.74 ± 0.07	1.63	1.60 ± 0.05	1.56	1.91 ± 0.07	1.94	1.49 ± 0.07	1.25*
BW40 (octaves)	1.88 ± 0.08	1.84	1.82 ± 0.06	1.81	1.96 ± 0.08	1.94	1.77 ± 0.07	1.69
Onset latency (ms)	13.6 ± 0.2	13	13.1 ± 0.2	12	13.2 ± 0.2	12	13.3 ± 0.3	12
Peak latency (ms)	21.4 ± 0.4	21	21.5 ± 0.4	21	21.9 ± 0.3	22	22.5 ± 1.0	21
End of peak latency (ms)	43.4 ± 1.2	40	47.9 ± 1.2	43*	50.6 ± 1.6	46*	54.9 ± 2.7	42*
Spont (Hz)	8.9 ± 0.6	7.5	10.3 ± 1.1	5.9	9.2 ± 0.6	8	5.6 ± 0.5	2.8*

Bolded and starred values are significantly different compared with control rats (Kruskal-Wallis non-parametric tests with *post-hoc* pairwise comparisons with Bonferroni correction).

## Discussion

Our previous study paired the sounds ‘rad’ and ‘lad’ with VNS 300 times per day and observed A1 plasticity specific to the paired sounds. In this study, we first replicated those findings. Vagus nerve stimulation was paired with speech sounds for 20 days, and neural responses were then recorded from the primary auditory cortex. Pairing the sounds ‘rad’ and ‘lad’ with VNS increased the A1 response strength and neural discriminability of the paired sounds. Additionally, we were able to expand on our previous findings to document that pairing ‘rad’ but not ‘lad’ with VNS did not alter A1 responses to the presented speech sounds. Pairing the sound ‘rad’ while ‘lad’ was an unpaired background sound did not result in A1 response strength or neural discriminability changes. These results indicate that the sounds that are presented and paired with VNS influence the neural plasticity observed following VNS-sound pairing.

### VNS-sound pairing parameters

We varied the number of VNS-sound pairings presented per day to determine if a larger number of pairings are needed to produce greater neural plasticity. While most previous VNS-sound pairing plasticity studies performed 300 VNS-sound pairings per day for 20 days (Engineer et al., 2011, 2015a, 2017b; Shetake et al., 2012; Borland et al., 2015, 2019; Loerwald et al., 2017; Buell et al., 2018, 2019; Adcock et al., 2020), one study performed 50 VNS-sound pairings per day for 20 days and observed no plasticity (Borland et al., 2018). In this study, we examined neural responses after 300 or 600 VNS-sound pairings per day and found that 600 VNS-sound pairings per day does not provide additional plasticity benefits. 300 VNS-sound pairings per day is sufficient to strengthen neural responses and make neural responses to individual sounds more discriminable. This suggests that more VNS-sound pairing is not necessarily better. Other studies examining the intensity, frequency, inter-stimulus interval, number of pulses, and duration of VNS indicate that there is an inverted-U function where too little or too much stimulation does not result in plasticity (Borland et al., 2015, 2018; Buell et al., 2018, 2019). For example, a stimulation intensity of 0.8 mA consistently generates plasticity specific to the paired sound (Engineer et al., 2011, 2015a; Adcock et al., 2020), while less stimulation intensity (0.2 mA) or more stimulation intensity (1.6 mA) does not generate plasticity, generating an inverted-U shaped result, where too little or too much intensity is ineffective and a moderate intensity is needed to generate stimulus-specific plasticity (Borland et al., 2015). Future studies are necessary to determine the optimal number of VNS-sound pairings per day to generate plasticity.

### Sound details play a key role in VNS-sound pairing

Similarly, the sounds that are paired with VNS play a large role in the observed plasticity (Lai and David, 2021). For example, previous studies have documented that pairing VNS with a 9 kHz tone results in plasticity specific to frequencies surrounding 9 kHz, while pairing VNS with a 19 kHz tone results in plasticity specific to frequencies surrounding 19 kHz (Engineer et al., 2011). In this study, pairing both ‘rad’ and ‘lad’ with VNS resulted in plasticity specific to the paired sounds, while pairing ‘rad’ but not ‘lad’ did not result in paired sound specific plasticity. Previous studies have also documented that background sounds influence cortical plasticity (Ohl and Scheich, 1996; Kilgard et al., 2001; Moucha et al., 2005). For example, nucleus basalis stimulation paired with frequency modulated (FM) sweeps of sounds spanning the rat hearing range did not alter A1 responses (Moucha et al., 2005). However, when the same FM sweeps were paired with nucleus basalis stimulation in the presence of randomly interleaved acoustically distinct unpaired background sweeps differing in sweep rate or FM direction, A1 responses were significantly altered.

The spectral and temporal acoustics of the paired and unpaired sound likely play a role in plasticity outcomes. The sounds ‘rad’ and ‘lad’ are acoustically very similar [differing primarily in the third formant transition (Miyawaki et al., 1975)] and activate the same populations of neurons, which may have caused any potential plasticity to the sound ‘rad’ to be canceled out when ‘lad’ was an unpaired background sound. Performing the same experiment but with sound pairs that are very acoustically distinct from each other and that evoke a more distinct neural response would likely lead to a different plasticity outcome. A previous study found that pairing a tone with nucleus basalis (NB) stimulation resulted in a 20% increase in the receptive field size of primary auditory cortex neurons. Interestingly, pairing the same tone with NB stimulation while tones of flanking frequencies were presented but not paired with NB stimulation did not result in any changes to A1 receptive field size (Kilgard et al., 2001). A study in the somatosensory system found that behaviorally important stimuli enlarge the population of responsive cells, while irrelevant stimuli shrink the population of responsive cells (Rabinovich et al., 2022). Since ‘rad’ and ‘lad’ activate the same population of cells in our study, it is possible that pairing ‘rad’ with neuromodulator release makes it behaviorally important, while not pairing ‘lad’ with neuromodulator release makes it irrelevant, and as a result, ‘lad’ canceled out any potential plasticity to ‘rad’. A better understanding of how auditory networks learn to extract subtle, but important, acoustic information from speech could be valuable for enhancing auditory processing in multiple populations, including second language learning, neurodevelopmental disorders, and recovery from aphasia.

### Neural response strength does not predict neural discriminability

One interesting finding in the current study was that the strength of the A1 response to the paired sounds does not predict how discriminable the sounds are. For example, pairing VNS with ‘rad’ and ‘lad’ 300 times per day leads to stronger A1 responses (Figure 2) and more discriminable A1 responses (Figure 4). Pairing VNS with ‘rad’ and ‘lad’ 600 times per day leads to stronger A1 responses (Figure 2) but does not alter A1 discriminability (Figure 4). This finding is consistent with a previous study that found that more spikes does not necessarily translate to more discriminable neural activity patterns (Raizada et al., 2010).

We have previously documented that A1 neural activity pattern discriminability accurately predicts behavioral discrimination ability (Engineer et al., 2008). Sounds with very similar neural activity patterns were difficult for rats to discriminate behaviorally, while sounds with very distinct neural activity patterns were easy for rats to discriminate behaviorally. Future studies are needed to evaluate the behavioral outcomes after each of the tested VNS-pairing conditions.

Interestingly, the increase in the response strength to the paired sounds did not generalize to an increase in response strength to tones in the VNS + RL paired group. Rats who received VNS paired with ‘rad’ and ‘lad’ 300 times per day had an increased response strength to the paired sounds, but an unaltered response strength to tones, which were not presented or paired with VNS. In contrast, rats who received VNS paired with ‘rad’ and ‘lad’ 600 times per day had an increased response strength to the paired sounds as well as an increased response strength to tones. Finally, rats who received VNS paired with ‘rad’ but not ‘lad’ had no response strength alterations to the paired sounds or to tones. This suggests that while more pairings did not produce greater neural plasticity to the paired sounds, 600 VNS-sound pairings per day resulted in plasticity that generalized to sounds in addition to the paired sounds. Previous studies have documented a generalization of VNS-pairing benefits to stimuli in addition to the paired stimuli (Meyers et al., 2018; Noble et al., 2018; Souza et al., 2021).

All of the VNS-sound paired groups exhibited a consistent increase in the A1 end of peak latency evoked by sounds (Table 2). As there were no alterations in the onset latency or peak latency evoked by sounds in any of the VNS-sound paired groups, this indicates that sounds evoke a longer A1 response in all VNS-sound paired groups. While the receptive field parameters in the VNS + RL paired groups remained stable, the VNS + R group had smaller receptive field bandwidths 20 and 30 dB above threshold, indicating that A1 in these rats responds to a narrower range of frequencies compared to experimentally naïve control rats. The VNS + R group additionally had a significantly weaker spontaneous firing rate, indicating that A1 in these rats was less excitable compared to experimentally naïve control rats. Multiple patient populations experience hypersensitivity to sounds, and this VNS-sound pairing strategy of pairing one sound with VNS while presenting, but not pairing, another acoustically similar sound, might be a beneficial way to reduce hypersensitivity to sound.

### The time course of plasticity

Multiple previous studies have documented that the auditory cortex changes observed depend on when during the learning process plasticity is measured (Fritz et al., 2005; Reed et al., 2011; Takahashi et al., 2011). Primary auditory cortex responses have been observed to change during perceptual learning, and renormalize during later stages of learning. This expansion-renormalization model of plasticity and learning could potentially help explain our finding that the VNS + R group had significant A1 receptive field changes yet no A1 response strength or neural discriminability changes to the paired sounds, while the VNS + RL and VNS + RL600 groups did not exhibit significant A1 receptive field changes. Additional studies are also needed to examine the time course of plasticity changes.

### The neuromodulatory systems activated by VNS

Stimulation of the vagus nerve activates multiple neuromodulatory systems, including acetylcholine, norepinephrine, dopamine, and serotonin (Nichols et al., 2011; Hulsey et al., 2016, 2017, 2019; Collins et al., 2021; Bowles et al., 2022; Rodenkirch et al., 2022). Many previous studies have focused on directly activating individual neuromodulatory systems and have observed stimulus-specific plasticity after pairing a sound with neuromodulator activation (Bakin and Weinberger, 1996; Kilgard and Merzenich, 1998). For example, pairing acetylcholine (ACh) release, through stimulation of the nucleus basalis, with the presentation of a tone results in an enhanced response to the paired tone frequency in the inferior colliculus (IC) and primary auditory cortex (A1) (Ma and Suga, 2003; Zhang and Suga, 2005; Froemke et al., 2013; Martins and Froemke, 2015). Similarly, pairing norepinephrine (NE) release, through stimulation of the locus coeruleus, with the presentation of a tone strengthens the neural response to the paired tone in IC, the medial geniculate nucleus of the thalamus, and A1 (Edeline et al., 2011; Martins and Froemke, 2015; Glennon et al., 2018). Determining exactly which neuromodulatory systems are activated by VNS and how they are activated is likely important for determining VNS plasticity mechanisms, and this likely is also important clinically. Neuromodulatory release driven by VNS enhances synaptic plasticity in networks that are specifically engaged by rehabilitation. Both the details of neuromodulatory release patterns as well as the details of neural activity driven by sensory or motor events are important in generating plasticity (Frémaux and Gerstner, 2015; Gerstner et al., 2018; Darrow et al., 2021). Future studies are needed to understand how the pattern of neural activity paired with neuromodulator release produces plasticity, in order to optimize the beneficial effects of VNS on therapeutic plasticity and rehabilitation. For individuals with auditory processing problems and hyporesponsive neural responses to sounds, VNS paired with sounds could represent a new therapeutic avenue that could lead to improved auditory processing of sounds.

## Data availability statement

The raw data supporting the conclusions of this article will be made available by the authors, without undue reservation.

## Ethics statement

The animal study was approved by The University of Texas at Dallas IACUC. The study was conducted in accordance with the local legislation and institutional requirements.

## Author contributions

MB: conceptualization, methodology, software, data curation, investigation, supervision, and writing – review and editing. EB, AC, NM, KG, and JB: investigation, and writing – review and editing. JR: formal analysis, visualization, and writing – review and editing. PS: supervision, investigation, and writing – review and editing. MK: conceptualization, and writing – review and editing. CE: conceptualization, software, data curation, formal analysis, funding acquisition, visualization, project administration, and writing – original draft. All authors contributed to the article and approved the submitted version.

## Funding

This research was supported by the National Institutes of Health R01DC017480 (CE).

## Conflict of interest

MK is a paid consultant for MicroTransponder Inc., and CE is married to an employee of MicroTransponder Inc.

The remaining authors declare that the research was conducted in the absence of any commercial or financial relationships that could be construed as a potential conflict of interest.

## Publisher’s note

All claims expressed in this article are solely those of the authors and do not necessarily represent those of their affiliated organizations, or those of the publisher, the editors and the reviewers. Any product that may be evaluated in this article, or claim that may be made by its manufacturer, is not guaranteed or endorsed by the publisher.

## References

[ref1] Adcock K. S. Chandler C. Buell E. P. Solorzano B. R. Loerwald K. W. Borland M. S.. (2020). Vagus nerve stimulation paired with tones restores auditory processing in a rat model of Rett syndrome. Brain Stimul. 13, 1494–1503. doi: 10.1016/j.brs.2020.08.006, PMID: 32800964

[ref2] Bakin J. S. Weinberger N. M. (1996). Induction of a physiological memory in the cerebral cortex by stimulation of the nucleus basalis. Proc. Natl. Acad. Sci. U. S. A. 93, 11219–11224. doi: 10.1073/pnas.93.20.11219, PMID: 8855336 PMC38311

[ref3] Borland M. S. Engineer C. T. Vrana W. A. Moreno N. A. Engineer N. D. Vanneste S.. (2018). The interval between VNS-tone pairings determines the extent of cortical map plasticity. Neuroscience 369, 76–86. doi: 10.1016/j.neuroscience.2017.11.004, PMID: 29129793 PMC5766390

[ref4] Borland M. S. Vrana W. A. Moreno N. A. Fogarty E. A. Buell E. P. Sharma P.. (2015). Cortical map plasticity as a function of vagus nerve stimulation intensity. Brain Stimul. 9, 117–123. doi: 10.1016/j.brs.2015.08.018, PMID: 26460200 PMC4724352

[ref5] Borland M. S. Vrana W. A. Moreno N. A. Fogarty E. A. Buell E. P. Vanneste S.. (2019). Pairing vagus nerve stimulation with tones drives plasticity across the auditory pathway. J. Neurophysiol. 122, 659–671. doi: 10.1152/jn.00832.2018, PMID: 31215351 PMC6734404

[ref6] Bowles S. Hickman J. Peng X. Williamson W. R. Huang R. Washington K.. (2022). Vagus nerve stimulation drives selective circuit modulation through cholinergic reinforcement. Neuron 110, 2867–2885.e7. doi: 10.1016/j.neuron.2022.06.017 35858623 PMC10212211

[ref7] Buell E. P. Borland M. S. Loerwald K. W. Chandler C. Hays S. A. Engineer C. T.. (2019). Vagus nerve stimulation rate and duration determine whether sensory pairing produces neural plasticity. Neuroscience 406, 290–299. doi: 10.1016/j.neuroscience.2019.03.019, PMID: 30904665 PMC6511481

[ref8] Buell E. P. Loerwald K. W. Engineer C. T. Borland M. S. Buell J. M. Kelly C. A.. (2018). Cortical map plasticity as a function of vagus nerve stimulation rate. Brain Stimul. 11, 1218–1224. doi: 10.1016/j.brs.2018.07.045, PMID: 30037658 PMC6487479

[ref9] Callan D. E. Tajima K. Callan A. M. Kubo R. Masaki S. Akahane-Yamada R. (2003). Learning-induced neural plasticity associated with improved identification performance after training of a difficult second-language phonetic contrast. NeuroImage 19, 113–124. doi: 10.1016/S1053-8119(03)00020-X, PMID: 12781731

[ref10] Collins L. Boddington L. Steffan P. J. McCormick D. (2021). Vagus nerve stimulation induces widespread cortical and behavioral activation. Curr. Biol. 31, 2088–2098.e3. doi: 10.1016/j.cub.2021.02.049 33740425

[ref11] Darrow M. J. Mian T. M. Torres M. Haider Z. Danaphongse T. Seyedahmadi A.. (2021). The tactile experience paired with vagus nerve stimulation determines the degree of sensory recovery after chronic nerve damage. Behav. Brain Res. 396:112910. doi: 10.1016/j.bbr.2020.112910, PMID: 32971197 PMC7572822

[ref12] Edeline J.-M. Manunta Y. Hennevin E. (2011). Induction of selective plasticity in the frequency tuning of auditory cortex and auditory thalamus neurons by locus coeruleus stimulation. Hear. Res. 274, 75–84. doi: 10.1016/j.heares.2010.08.005, PMID: 20709165

[ref13] Engineer C. T. Engineer N. D. Riley J. R. Seale J. D. Kilgard M. P. (2015a). Pairing speech sounds with vagus nerve stimulation drives stimulus-specific cortical plasticity. Brain Stimul. 8, 637–644. doi: 10.1016/j.brs.2015.01.408, PMID: 25732785 PMC4461522

[ref14] Engineer C. T. Hays S. A. Kilgard M. P. (2017a). Vagus nerve stimulation as a potential adjuvant to behavioral therapy for autism and other neurodevelopmental disorders. J. Neurodev. Disord. 9:20. doi: 10.1186/s11689-017-9203-z, PMID: 28690686 PMC5496407

[ref15] Engineer N. D. Percaccio C. R. Pandya P. K. Moucha R. Rathbun D. L. Kilgard M. P. (2004). Environmental enrichment improves response strength, threshold, selectivity, and latency of auditory cortex neurons. J. Neurophysiol. 92, 73–82. doi: 10.1152/jn.00059.2004, PMID: 15014105

[ref16] Engineer C. T. Perez C. A. Chen Y. H. Carraway R. S. Reed A. C. Shetake J. A.. (2008). Cortical activity patterns predict speech discrimination ability. Nat. Neurosci. 11, 603–608. doi: 10.1038/nn.2109, PMID: 18425123 PMC2951886

[ref17] Engineer C. T. Rahebi K. C. Buell E. P. Fink M. K. Kilgard M. P. (2015b). Speech training alters consonant and vowel responses in multiple auditory cortex fields. Behav. Brain Res. 287, 256–264. doi: 10.1016/j.bbr.2015.03.044, PMID: 25827927 PMC4424170

[ref18] Engineer N. D. Riley J. R. Seale J. D. Vrana W. A. Shetake J. A. Sudanagunta S. P.. (2011). Reversing pathological neural activity using targeted plasticity. Nature 470, 101–104. doi: 10.1038/nature09656 21228773 PMC3295231

[ref19] Engineer C. T. Shetake J. A. Engineer N. D. Vrana W. A. Wolf J. T. Kilgard M. P. (2017b). Temporal plasticity in auditory cortex improves neural discrimination of speech sounds. Brain Stimul. 10, 543–552. doi: 10.1016/j.brs.2017.01.007, PMID: 28131520 PMC5410401

[ref20] Frémaux N. Gerstner W. (2015). Neuromodulated spike-timing-dependent plasticity, and theory of three-factor learning rules. Front. Neural Circuits 9:85. doi: 10.3389/fncir.2015.00085, PMID: 26834568 PMC4717313

[ref21] Fritz J. B. Elhilali M. Shamma S. A. (2005). Differential dynamic plasticity of A1 receptive fields during multiple spectral tasks. J. Neurosci. 25, 7623–7635. doi: 10.1523/JNEUROSCI.1318-05.2005, PMID: 16107649 PMC6725393

[ref22] Froemke R. C. Carcea I. Barker A. J. Yuan K. Seybold B. A. Martins A. R. O.. (2013). Long-term modification of cortical synapses improves sensory perception. Nat. Neurosci. 16, 79–88. doi: 10.1038/nn.3274 23178974 PMC3711827

[ref23] Froemke R. C. Merzenich M. M. Schreiner C. E. (2007). A synaptic memory trace for cortical receptive field plasticity. Nature 450, 425–429. doi: 10.1038/nature06289, PMID: 18004384

[ref24] Gerstner W. Lehmann M. Liakoni V. Corneil D. Brea J. (2018). Eligibility traces and plasticity on behavioral time scales: experimental support of NeoHebbian three-factor learning rules. Front. Neural Circuits 12:53. doi: 10.3389/fncir.2018.00053, PMID: 30108488 PMC6079224

[ref25] Glennon E. Carcea I. Martins A. R. O. Multani J. Shehu I. Svirsky M. A.. (2018). Locus coeruleus activation accelerates perceptual learning. Brain Res. 1709, 39–49. doi: 10.1016/j.brainres.2018.05.048, PMID: 29859972 PMC6274624

[ref26] Hulsey D. R. Hays S. A. Khodaparast N. Ruiz A. Das P. Rennaker R. L.. (2016). Reorganization of motor cortex by Vagus nerve stimulation requires cholinergic innervation. Brain Stimul. 9, 174–181. doi: 10.1016/j.brs.2015.12.007, PMID: 26822960 PMC4789078

[ref27] Hulsey D. R. Riley J. R. Loerwald K. W. Rennaker R. L. Kilgard M. P. Hays S. A. (2017). Parametric characterization of neural activity in the locus coeruleus in response to vagus nerve stimulation. Exp. Neurol. 289, 21–30. doi: 10.1016/j.expneurol.2016.12.005, PMID: 27988257 PMC5297969

[ref28] Hulsey D. R. Shedd C. M. Sarker S. F. Kilgard M. P. Hays S. A. (2019). Norepinephrine and serotonin are required for vagus nerve stimulation directed cortical plasticity. Exp. Neurol. 320:112975. doi: 10.1016/j.expneurol.2019.112975, PMID: 31181199 PMC6708444

[ref29] Kilgard M. P. Merzenich M. M. (1998). Cortical map reorganization enabled by nucleus basalis activity. Science 279, 1714–1718. doi: 10.1126/science.279.5357.1714. 9497289

[ref30] Kilgard M. P. Pandya P. K. Vazquez J. Gehi A. Schreiner C. E. Merzenich M. M. (2001). Sensory input directs spatial and temporal plasticity in primary auditory cortex. J. Neurophysiol. 86, 326–338. doi: 10.1152/jn.2001.86.1.326, PMID: 11431514

[ref31] Kuhl P. K. (2004). Early language acquisition: cracking the speech code. Nat. Rev. Neurosci. 5, 831–843. doi: 10.1038/nrn1533, PMID: 15496861

[ref32] Lai J. David S. V. (2021). Short-term effects of vagus nerve stimulation on learning and evoked activity in auditory cortex. eNeuro 8:ENEURO.0522-20.2021. doi: 10.1523/eneuro.0522-20.2021, PMID: 34088737 PMC8240839

[ref33] Loerwald K. W. Borland M. S. Rennaker R. L. Hays S. A. Kilgard M. P. (2017). The interaction of pulse width and current intensity on the extent of cortical plasticity evoked by vagus nerve stimulation. Brain Stimul. 11, 271–277. doi: 10.1016/j.brs.2017.11.007, PMID: 29174302 PMC5898968

[ref34] Logan J. S. Lively S. E. Pisoni D. B. (1991). Training Japanese listeners to identify English /r/ and /l/: a first report. J. Acoust. Soc. Am. 89, 874–886. doi: 10.1121/1.1894649, PMID: 2016438 PMC3518834

[ref35] Ma X. Suga N. (2003). Augmentation of plasticity of the central auditory system by the basal forebrain and/or somatosensory cortex. J. Neurophysiol. 89, 90–103. doi: 10.1152/jn.00968.2001 12522162

[ref36] Martins A. R. O. Froemke R. C. (2015). Coordinated forms of noradrenergic plasticity in the locus coeruleus and primary auditory cortex. Nat. Neurosci. 18, 1483–1492. doi: 10.1038/nn.4090, PMID: 26301326 PMC4583810

[ref37] Meyers E. C. Solorzano B. R. James J. Ganzer P. D. Lai E. S. Rennaker R. L.. (2018). Vagus nerve stimulation enhances stable plasticity and generalization of stroke recovery. Stroke 49, 710–717. doi: 10.1161/STROKEAHA.117.019202, PMID: 29371435 PMC6454573

[ref38] Miyawaki K. Jenkins J. J. Strange W. Liberman A. M. Verbrugge R. Fujimura O. (1975). An effect of linguistic experience: the discrimination of [r] and [l] by native speakers of Japanese and English. Percept. Psychophys. 18, 331–340. doi: 10.3758/BF03211209

[ref39] Moucha R. Pandya P. K. Engineer N. D. Rathbun D. L. Kilgard M. P. (2005). Background sounds contribute to spectrotemporal plasticity in primary auditory cortex. Exp. Brain Res. 162, 417–427. doi: 10.1007/s00221-004-2098-4, PMID: 15616812 PMC2950066

[ref40] Nichols J. A. Nichols A. R. Smirnakis S. M. Engineer N. D. Kilgard M. P. Atzori M. (2011). Vagus nerve stimulation modulates cortical synchrony and excitability through the activation of muscarinic receptors. Neuroscience 189, 207–214. doi: 10.1016/j.neuroscience.2011.05.024, PMID: 21627982

[ref41] Noble L. J. Meruva V. B. Hays S. A. Rennaker R. L. Kilgard M. P. McIntyre C. K. (2018). Vagus nerve stimulation promotes generalization of conditioned fear extinction and reduces anxiety in rats. Brain Stimul. 12, 9–18. doi: 10.1016/j.brs.2018.09.013, PMID: 30287193 PMC6301121

[ref42] Ohl F. W. Scheich H. (1996). Differential frequency conditioning enhances spectral contrast sensitivity of units in auditory cortex (field Al) of the alert Mongolian gerbil. Eur. J. Neurosci. 8, 1001–1017. doi: 10.1111/j.1460-9568.1996.tb01587.x 8743748

[ref43] Perez C. A. Engineer C. T. Jakkamsetti V. Carraway R. S. Perry M. S. Kilgard M. P. (2013). Different timescales for the neural coding of consonant and vowel sounds. Cereb. Cortex 23, 670–683. doi: 10.1093/cercor/bhs045, PMID: 22426334 PMC3563339

[ref44] Rabinovich R. J. Kato D. D. Bruno R. M. (2022). Learning enhances encoding of time and temporal surprise in mouse primary sensory cortex. Nat. Commun. 13:5504. doi: 10.1038/s41467-022-33141-y, PMID: 36127340 PMC9489862

[ref45] Raizada R. D. S. Tsao F.-M. Liu H.-M. Kuhl P. K. (2010). Quantifying the adequacy of neural representations for a cross-language phonetic discrimination task: prediction of individual differences. Cereb. Cortex 20, 1–12. doi: 10.1093/cercor/bhp076, PMID: 19386636 PMC2860710

[ref46] Recanzone G. H. Schreiner C. E. Merzenich M. M. (1993). Plasticity in the frequency representation of primary auditory cortex following discrimination training in adult owl monkeys. J. Neurosci. 13, 87–103. doi: 10.1523/JNEUROSCI.13-01-00087.1993, PMID: 8423485 PMC6576321

[ref47] Reed A. Riley J. Carraway R. Carrasco A. Perez C. Jakkamsetti V.. (2011). Cortical map plasticity improves learning but is not necessary for improved performance. Neuron 70, 121–131. doi: 10.1016/j.neuron.2011.02.038 21482361

[ref48] Rios M. Bucksot J. Rahebi K. Engineer C. Kilgard M. Hays S.. (2019). Protocol for construction of rat nerve stimulation cuff electrodes. Methods Protoc. 2:19. doi: 10.3390/mps2010019, PMID: 30957053 PMC6448795

[ref49] Rodenkirch C. Carmel J. B. Wang Q. (2022). Rapid effects of vagus nerve stimulation on sensory processing through activation of neuromodulatory systems. Front. Neurosci. 16, 1–14. doi: 10.3389/fnins.2022.922424 PMC9294458 35864985

[ref50] Shetake J. A. Engineer N. D. Vrana W. A. Wolf J. T. Kilgard M. P. (2012). Pairing tone trains with vagus nerve stimulation induces temporal plasticity in auditory cortex. Exp. Neurol. 233, 342–349. doi: 10.1016/j.expneurol.2011.10.026, PMID: 22079155

[ref51] Souza R. R. Oleksiak C. R. Tabet M. N. Rennaker R. L. Hays S. A. Kilgard M. P.. (2021). Vagus nerve stimulation promotes extinction generalization across sensory modalities. Neurobiol. Learn. Mem. 181:107425. doi: 10.1016/j.nlm.2021.107425, PMID: 33771710 PMC12060723

[ref52] Takahashi H. Yokota R. Funamizu A. Kose H. Kanzaki R. (2011). Learning-stage-dependent, field-specific, map plasticity in the rat auditory cortex during appetitive operant conditioning. Neuroscience 199, 243–258. doi: 10.1016/j.neuroscience.2011.09.046, PMID: 21985937

[ref53] Yokota R. Aihara K. Kanzaki R. Takahashi H. (2015). Learning-stage-dependent plasticity of temporal coherence in the auditory cortex of rats. Brain Topogr. 28, 401–410. doi: 10.1007/s10548-014-0359-5, PMID: 24615394

[ref54] Zhang Y. Suga N. (2005). Corticofugal feedback for collicular plasticity evoked by electric stimulation of the inferior colliculus. J. Neurophysiol. 94, 2676–2682. doi: 10.1152/jn.00549.2005, PMID: 16000518

